# The sport volunteering paradox: concerns from a legal and governance perspective, but the irony remains

**DOI:** 10.3389/fspor.2026.1746196

**Published:** 2026-04-22

**Authors:** Charles Mountifield, Stirling Sharpe

**Affiliations:** 1 UC Research Institute for Sport and Exercise (UCRISE), University of Canberra, Canberra, ACT, Australia; 2 Discipline of Sport and Exercise Science, Faculty of Health, University of Canberra, Canberra, ACT, Australia

**Keywords:** employment, governance, insurance, sport, volunteers

## Abstract

In Australia, volunteers represent a ubiquitous—and critical—feature of organised sport. Sporting events, be they the Olympic Games or a grassroots football match, require the services of a human resource whose engagement, in turn, necessitates consideration from a legal and governance standpoint. For the organisations administering sporting activities, however, volunteers represent an anomaly in an hierarchical sport system. Volunteers are often subject to the whim and caprice of institutions deficient in processes for engaging an unpaid workforce. Issues concerning employment terms and volunteer rights have proved contentious, especially in the community sport sector. Although volunteer numbers have fluctuated, legal problems ensue, resulting in various “injuries” and other considerations relating to the treatment of sport volunteers. There is a dilemma for sport organisations: the governance model for community sport is flawed due to the paradox between top-down policy edicts and the ability of volunteers to manage themselves by virtue of their non-professional roles. By way of a narrative literature review, this paper identifies issues impacting sport organisations and points to the irony that volunteers are unlikely to be able to fulfil expectations based on existing governance structures.

## Introduction

Volunteers in Australian sport are a vital organisational resource, from mega-events to grassroots pursuits (1–3). Managing volunteers is an essential and demanding aspect for the architects of sport policy (4, 5) and there are various circumstantial issues and long-term challenges that impact sport organisations (6, 7). Good governance in sport includes consideration of the volunteering sector (8), and sound management and governance are increasingly on the agenda of governments and sport organisations (8). The contextual concerns, however—including conceptual and operational transparency and stability (9)—make it difficult to effectively define and apply universal principles of “good” governance to sport organisations at all levels (10–12). This situation, in turn, gives rise to legal complexities, including issues related to the duty of care that sport organisations owe to various stakeholders (13–15). Indeed, from a governance, policy, and legal perspective, there are various issues for sport organisations that are impacted by the practicalities of the organisation of community sport and the operation of community sport clubs (CSCs) (11, 14, 16). Arriving at a deeper understanding of the complexities of managing volunteer workforces involved with community sport will reduce the potential for legal issues and help reduce the sport volunteering paradox.

## Background and research objective

Community sport is the foundation of sporting activity and although it can have “multifarious meanings and diverse applications in different parts of the world” [(17), p. 1], community sport, or grassroots sport as it is often called, is defined as the elementary layer of sport (18) taking place at the community level (19). Community sport is invariably organised competitive physical activity provided by a CSC within a local setting (20). CSCs—a “cluster of competitive, but not high-performance sports” (21), p. 400)—have a significant role in providing organised sport for the wider population (22, 23). CSCs are run by volunteers who occupy positions such as coach, official, manager, administrator, board and committee member (24). In Australia, in 2022, approximately 90% of the 3.4 million Australians involved in non-participatory roles in sport and recreation were volunteers (25). Crucially, volunteers make significant contributions to sport's social and economic impact, especially in community settings (26).

Set against this backdrop, however, is evidence of numerous complexities associated with sport policy implementation at the community level (4, 27, 28). Indeed, on a macro basis, it is suggested that the community sport policy sector is conflicted, disorganised (29–32), and “fragmented, fractious and ineffective” (33), (p. 2). From the perspective of CSCs and policy implementation, there are many vagaries of managing volunteers in keeping with sound governance and associated policy development tenets. Some concerns relate to, for example, the nature of employment and the rights of volunteers, alongside health and safety issues in general. In addition, there are significant matters relating to decreasing volunteer numbers (34–36) and the capacity—including skills and expertise—that volunteers require in often demanding roles (22, 37, 38), an issue magnified in sport due to the high percentage of volunteers involved [e.g., (39)]. Such concerns present a paradox involving government and National Sporting Organisations (NSO) policy objectives (40), the reality of volunteer engagement in sport (3), and sport governance considerations (41).

Historically, sport governance systems were essentially an afterthought in Australia (42) and governance policy was erratic and divisive (8). This situation was not helped by the Australian federal political structure, where each state government adopted its own approach to governance, alongside NSOs that produce individual, sport-specific governance models (8). Moreover, the recent shift toward more professionalised governance structures has contributed to the partial alienation of CSCs (43), which are predominantly operated by volunteers (44). Top-down policy initiatives largely drive this trend (45–47), placing increasing demands on volunteer board members. In many CSCs, these individuals not only serve on the committee but also take on multiple roles, including coaching and officiating [e.g., (48, 49)]. The pressure to professionalise such amateur organisations reduces the time volunteers can dedicate to core sporting activities and shifts their focus toward administrative responsibilities. Additionally, meeting new governance expectations often requires further training, which may discourage others from stepping forward to help. When all this is considered in conjunction with the fact that volunteer hours are decreasing [e.g., (49, 50)] –, the professionalisation of the governance of CSCs may actually be detrimental to the on-field experience of participants as valuable volunteering hours are inversely dedicated to administrative tasks.

Within the challenging governance environment, representing a comparable afterthought in Australia, the universal management of volunteers in sport has lacked adequate policy considerations. Such circumstances apply to mainstream sport volunteering (5, 51) and have been observed recently concerning female involvement in community sport volunteering (52) and also the involvement of First Nations people in Australian sport volunteering in general (53). In that context, paradoxes in the volunteer sector are nothing new.Where volunteering in sport is concerned, there is an apparent contradiction with managing volunteers, notably relating to trends in how employment considerations impact volunteering (54). In addition, while sport governance may be an increasingly important area of focus for sport organisations, the reality is a somewhat ironic scenario because the majority of directors of sport organisations globally are volunteers (42, 55). Further, developments in sport governance internationally have created paradoxes that relate to attempts at dispersing power from national governance systems to the reality of centrally managed, monitored and controlled top-down policy delivery (18, 56).

In an effort to blend governance and the management of volunteers, some processes proved counterproductive to achieving operational objectives. In particular, there was a failure to deliver sports policy nearer to the end user and with relative autonomy from government, which appears, paradoxically, to be more in control, thus perpetuating top-down policy processes (56, 57). As part of this complex background, the notion of power in top-down governance structures (58, 59) leads to a further dilemma; Sam & Jackson (2004), for example, pinpoint issues concerning integration in the sport policy process, arguing that a paradigm incorporating more significant rationalisation and assimilation for key stakeholders serves as the basis to reduce the marginalisation of volunteer-run CSCs.

Overall, the significant volunteer-based aspect of the community sport has led CSCs—policy implementers (60, 61)—down a more operational path at the expense of enacting practical strategic objectives based on sound governance principles (62). For CSCs, real-world concerns impact the capacity to act within the guidelines and constraints of governance principles and implement policy effectively (8, 63, 64). Thus, echoing Parent et al. (41), “better” governance for CSCs is a consideration for research to better understand issues impacting the operational capacity of CSCs. Within that overall contextual framework—and the fundamental understanding that there is an apparent irony connected to volunteers in community sport—the objective herein will be to identify issues impacting CSCs in Australia from a governance and legal perspective. With this objective in mind, the paper addresses the following research question: *What are examples of governance and legal concerns impacting volunteers in community sport?.*

## Method

To address the research question, a narrative review format was adopted. Narrative reviews are considered a rigorous but also flexible approach to knowledge synthesis (65) and serve to summarise aspects of a topic (66), provide a structure that introduces the subject, outline the development in a narrative format, and conclude with a discussion element (67). Narrative reviews reflect oft-utilised approaches to sport-related research (68–70), including issues impacting sport policy considerations (71, 72). For this paper, to address the research question, the objective was to provide a broad synopsis of governance and legal issues, and to point to gaps in knowledge, a key focus of narrative reviews (73), and highlight areas to explore with additional research.

### Data collection

Data was collected from peer-reviewed journal articles, academic book chapters, and “grey” literature through searches using Google Scholar and university libraries, the latter of which allowed for the use of specific search terms. The data was selected based on the authors' knowledge of the field, and the key search terms were sport volunteers, sport governance, risk in sport management, legal issues and sport volunteers, and COVID-19 and sport volunteers. In the case of “grey” literature, current sport governance policies were examined, as were legal cases from online court documents. No restrictions on timeframes were applied, and the number of sources relied upon for the analysis (*n* = 66) was considered sufficient to inform the research objective.

### Data analysis

In keeping with the relative subjectivity of narrative reviews (65), the data was evaluated by the authors and subsequently grouped into the following themes: (1) volunteer labour, (2) governance, (3) risk, (4) evidence, and (5) COVID-19. This thematic allocation was based on the authors' interpretation of findings relevant to the study and the desire to provide a “narrative” that informed the research objective and pointed toward the need for further research.

## Literature review

In keeping with the concept of a narrative and to provide a link between key aspects of the research objective, the review is divided into five sections, as follows: (1) volunteer labour, to provide a platform for the subject; (2) governance, to outline the historic and current nature of the management of sport; (3) risk, to point to the issues facing sporting organisations in relation to volunteers; (4) evidence, to highlight legal outcomes linked to risk.; and (5) COVID-19, which presented an additional and novel issue for governance considerations. In so doing, the review provides a sequential outline of key aspects of this paper.

### Volunteer labour

From an overall policy standpoint, there is an initial and significantly fundamental issue to consider. A distinct feature of the sport sector is the reliance on volunteer labour to develop and deliver sport programs (74)—volunteers are often termed the “backbone” of the Australian sport system (2, 45, 75). The impact of diminishing volunteer numbers, however, results in a human resource that often lacks appropriate skills and thus—in what amounts to *real-world* concerns—CSCs face constant challenges in adopting strategic plans, pursuing objectives, or implementing policy (16, 30, 45, 76). A fundamental concern is the dependence on volunteer labour, but with often unclear boundaries governing the performance of duties and aspects of risk (1, 77).

Sport volunteers need appropriate training to obtain the skills and knowledge required to work competently and in safety. Despite the passion sport volunteers often exude in association with their role (78, 79), they often lack governance-related skillsets or experience, let alone the ability to understand their responsibilities and expectations properly (8, 80). CSC boards and committees often comprise a wide range of professional skill sets, including unskilled trades, leading to a potential vacuum of professional competencies to meet complex administrative demands (81) and creates limitations in the governance expertise of a CSC committee due to a lack of knowledge and skills (82).

At higher levels of the sport system, board and committee members are often appointed and elected due to their skills and training in governance. It is more likely that individuals will fill vacant roles with the appropriate knowledge and expertise to deliver on strategic objectives (83). At the community sport level, however, coupled with volunteers supervising other volunteers, along with time and workload constraints, there is the propensity of risk from a governance perspective. With that context, sport organisations ought to consider governance models incorporating programs that include appropriate training, role assignment, and evaluation (74).

### Governance

Regardless of size, sport organisations like CSCs must have sound governance principles in place and abide by the obligations under the relevant government Act, as dictated by the Australian Sports Commission (84). Crucially, proper governance is essential to minimise the potential for litigation from potentially injurious events. Indeed, with increasing levels of professionalisation and associated commercial pressures, the need for heightened accountability, and the survival and legitimacy of sport organisations, sport governance is becoming progressively more critical (8, 42, 85). There has been a pronounced shift from volunteer-driven grassroots sport to professionally managed and delivered sport, notably supported by volunteers (8). Equally, for CSCs that volunteers administer, there is an increasing expectation of professionalism (86). In Australia, sporting organisations are increasingly being held accountable for creating and implementing sound and manageable governance structures, where it is clear that “governance is not only relevant to large national sporting organisations; it is important for all organisations in our sector” (87), (p. 4).

When evaluating governance processes in sport, it is essential to note the complex nature of legal obligations that CSCs owe to their volunteer workforce. The National Volunteer Guide 2025 outlines the key legal obligations CSCs owe their volunteers (88). The guide primarily provides an overview of the key legal issues involved in engaging and managing volunteers such as regulations that address workplace health and safety and workplace behaviour. In such environments, organisations must understand the legal distinction between volunteers, employees, and independent contractors. By way of example, under Australian Work Health and Safety (WHS) Laws, volunteers occupy the role of “worker” and thus should be afforded the same consideration as a paid employee (89).

Robust governance structures in sport rely, in part, on overall policy formulation (41, 90), yet, sport policy development has been fraught with inconsistencies (91, 92) and is very much the subject of top-down processes (60, 71). The issue of policy voids in sport has not escaped academic scrutiny, with recent research pointing to the need for greater consideration of volunteers involved with CSCs (56, 59) and indeed ways to facilitate such consideration (47). In the Australian sport landscape, the transition to clearer governance structures requires implementing volunteer management practices that maximise performance, satisfaction, and retention through the practical design of volunteer programs (74, 77). Regarding “performance”, it is crucial to ensure volunteers can perform their tasks whilst minimising risk from a governance perspective (7, 93). Therein, however, lies the issue that relates to increasing pressures in sport where policy development can fail to incorporate the nuances of community sport due to the top-down nature of the policy process (27, 56, 60, 94). Subsequent policy failure creates a burden for various stakeholders and increases the paradox linked to the governance of community sport (40, 95) and the associated risk and legal concerns (14).

### Risk

Managing stakeholder interests is reflected in the need to control risk and protect sport organisations, their members, and volunteers from harm and potential legal action (96). Such issues are not lost in academic circles and have been highlighted repeatedly in literature on professionalisation linked to community sport (40, 86, 97, 98); it is a key point that underlines the difference between professional NSOs and volunteer-run CSCs (43). Within that complex structure, regulatory and compliance burdens on volunteers have increased. Along with more specific governance requirements, areas of concern include child safety, food safety, risk management, and member privacy and protection (1). Such responsibilities are amplified for CSCs, run by volunteers, where there is no legal obligation (although it is a recommendation) to adhere to work, health and safety legislation (89). This point is worsened because the National Volunteer Guide see (88) is a lengthy and complicated document that requires significant time and expertise to comprehend and enact effectively, thus representing a challenge for CSCs (16, 93).

From the duty of care perspective, some risks relate to the responsibilities owed to potentially vulnerable parties. In relation to the duty of care to volunteers, the objective is to assess which party must take precautions against the risk of harm that might lead to the injury of another party (15). Where match officials are concerned, there are various examples of officials in many sports reporting verbal and physical abuse (99), with Australian Rules Football umpires pointing to examples of both verbal and physical abuse (100) and Association Football providing multiple incidents of abuse on a weekly basis (101). For professional match officials, there are various protections afforded to them by virtue of their employment, but for volunteer match officials, there is an issue relating to the duty of care. Both the volunteer and the sport organisation have a duty of care, with the latter obligated to provide, among other things, risk management protocols and a safe working environment for the volunteer (88). Paradoxically, the volunteer is obligated to, among other things, take reasonable care for their own safety, with training and accreditation courses providing guidance on conflict management.

In the context of considerations for NSOs, some concerns relate to increasing levels of professionalisation and a duty of care. For example, an NSO may have different operational units evolving through the professionalisation continuum at different rates. There is likely to be a very professionalised executive office, finance and business units, and a high-performance department, yet the officiating unit may still meet around the “kitchen table” (102). Such scenarios can lead to situations where one group in an organisation—e.g., high-performance athletes—are managed differently from another—e.g., match officials. This disparity in treatment of key stakeholders creates a potential risk to the organisation where, for example, different levels of human resources are allocated to deal with injuries to match officials compared to resources available for the athletes (103).

Developing the officiating example further, some situations give rise to an element of risk where unreasonable burdens are placed on officials. For instance, volunteer members of the Hockey ACT Umpiring and Technical Committee are responsible for the allocation of match officials to games, a role that takes approximately 20 h of work a week across all competitions, with many of their work hours concentrated on a Friday and Saturday (often late at night as umpires withdraw from their appointed game and must be replaced) (104). These same volunteers are also expected to umpire and coach other officials each weekend. From the perspective of events, such as national championships, there are examples of officials working for 13 or more hours per day, followed by performing other administrative duties at accommodation facilities following the end of the day's play, an issue peculiar to sport volunteering (105). Often, based on observations of the authors, it was noted that such facilities are temporary and may not meet WHS guidelines. Such scenarios are common in many sports and indeed, symptomatic of overworked volunteers, which can lead to issues such as “dropout” (106, 107) and mental well-being concerns (108). From a governance perspective, sport organisations should consider the risk of injury, and associated legal ramifications, to the officials, participants, or other stakeholders that are managed by overworked volunteers.

### Evidence

The responsibility for volunteer-run CSC operations is onerous and involves a level of risk (109–111). Most CSCs are incorporated as not-for-profit associations bound by the relevant state-based Associations Incorporation Acts. Volunteering for a CSC involves a certain level of accountability for participant activities and management procedures (80). Indeed, those managing CSCs are at risk of being held responsible for the conduct of those who represent the CSCs either voluntarily or in employment. With that comes the issue of potential volunteers thinking very seriously—an example of the *irony*—before accepting roles with CSCs, because the legal implications can be extreme in the event of legal action (110).

An initial example was a situation in New Zealand that helped form the view of Sam & Jackson (2004). There were issues relating to integration in the sport policy process, and a quandary arose concerning the number of regional sports trusts. The dilemma centred on two fundamental contradictions and paradoxes arising from this paradigm shift: the first being the historical issues that lead to disjointed sport delivery structures, such as governmental focus on regionalised control and the endorsement of contractual arrangements; the second being the nature and suitability of centralised regulation due to a clear need for independent and localised policy delivery. Such deliberations impact the function of key stakeholders—CSCs—who have limited input into policy creation (56, 59, 60, 112).

From the perspective of what constitutes employment—further example of the *irony*—the National Volunteer Guide [see (88)] is potentially confusing: merely labelling a worker a “volunteer”, “employee” or “independent contractor” does not mean they are a “volunteer”, an “employee” or an “independent contractor”. From an overarching legal view, such classifications have led to varied and problematic interpretations of the working relationship, compared to the title in the job description. As evidenced in *WorkPac Pty Ltd v Rossato* [2021] HCA 23–271 CLR 456, the court looked beyond the label to the substance of the professional association, noting that the word “casual” was not an accurate representation of the relationship. In a sporting example, where a CSC swimming coach gave a junior athlete a high-energy caffeine drink, the coach was punished under the association's disciplinary procedures. In a subsequent court ruling, however, it was found that the association had no authority to penalise the coach as the coach was deemed a contractor and not a member of the association (113).

Developing the matter of employment further, the decision of *Wieland v Return to Work SA* [2018] SAET 190 confirms that volunteers can be regarded as employees and attract the protection of relevant regulations. In this example, the court found that a volunteer coach was entitled to compensation for an injury because the individual was classified as an employee and protected by legislation for mishaps relating to the performance of duties. For CSCs, a concern arises in relation to how individuals—noting the aforementioned point on “labels” resulting from *WorkPac Pty Ltd v Rossato* [2021] HCA 23–271 CLR 456—who provide their services voluntarily are categorised. Such issue is highlighted due to the lack of a statutory definition of volunteer, such that courts and tribunals must will rely on previous cases to inform their view (114).

In *Grinholz v Football Federation Victoria Inc* [2016] FWC 7976, the Fair Work Commission considered if a volunteer coach was an employee protected from unfair dismissal by the Fair Work Act 2009 (Cth). In this example, the most significant consideration was that the claimant accepted an honorarium as consideration for the provision of services. In the proceedings, the claimant asked to be considered an employee who could not be unfairly dismissed. Although it was held that the claimant was a volunteer, the case creates potential issues because unfair dismissal claims could impact CSCs (115).

Concerning the matter of a duty of care, the issue for CSCs is challenging due to the reliance on volunteers and the often-unincorporated status of the CSC (which limits the parties for the injured party to sue). In the case of *Hrybynyuk v Mazur* [2004] NSWCA 374, the plaintiff was a member of an unincorporated association. While engaged as a volunteer, the plaintiff was injured in the performance of the duties. The plaintiff sued the club president, suggesting a duty of care was owed by the president. The court ruled that whilst there was a duty of care, there had been no breach of this duty. The findings in this case were instructive, especially for CSCs and the potential liability in negligence (133).

From a public and product liability standpoint, based on *Dann v Port Sorell Bowls Club Inc* [2020] TASSC 47, CSCs are required to advise volunteers not to handle mugs of burning oil. In this case, a CSC volunteer cooking at a BBQ successfully sued the CSC for negligence after suffering burns to his hand when removing a mug of burning oil from beneath the BBQ. The court found that the defendant should have taken several precautions to avoid the charge of negligence. For CSCs, the implications are significant, particularly given that volunteers facilitate such occasions and often rely on fundraising barbecues as a source of income (116). Similarly, organisers of food-based fundraisers need to ensure the CSC is compliant with food safety handling legislation (117). Compliance, in this case, comes at the expense of volunteer time to gain an accreditation.

### COVID-19

The advent of COVID-19 gave rise to numerous examples of legal considerations that, after the return to sport, meant participants and volunteers were faced with paradoxical challenges that revolved around various drawbacks linked to public health (54, 118). Indeed, there were numerous concerns about how to manage community sport in what were described as “turbulent times” (45), (p. 7) and created issues surrounding the duty of care (15). As one example in Australia, problems arose where potential volunteers for CSCs were not prepared to offer their services as Safety Coordinators due to the complexity of the role, which in turn brings with it the possible liability issues (119). In this example, a notable concern was the potential for the Safety Coordinator to be sued personally by third parties impacted directly by COVID-19 due to involvement with community sport. Whilst there is always the chance that a volunteer might act in bad faith or be grossly negligent to be personally liable, the return to sport created a risk for CSCs and potential liability for their committee members as well (119).

Many other instances impacted CSCs, evidenced in the numerous and often onerous legal guidelines for community organisations [e.g., (120)]. In particular, the range of obligations reflected the dynamic nature of the pandemic that hindered the development and implementation of a clear strategy for an unfettered return to mass participatory sport. Instead, constant modifications of regulations were introduced, which in turn led to chaos and incomprehension of ever-changing rules (121, 122). Overall, the processes were often counterproductive and led to resistance from CSCs to more formal, professional approaches to running community sport (86, 123). Such resistance is in sharp contrast to the edicts of top-down policy but is in keeping with the concept of the capacity of CSCs (22, 56, 124, 125).

## Discussion and future direction

The objective of this paper was to provide an outline of governance and legal concerns impacting community sport volunteers. Such outline considered the organisations (e.g., CSCs) that community sport volunteers are responsible for, and with which comes risk. The study was not meant to be exhaustive and instead, based on the observations in the literature, confirmed that a paradox is evident where volunteers in sport are concerned. Crucially, the range of case law does not adequately deal with issues connected to volunteers and issues such as the determination of specific roles and responsibilities, along with confusion arising from the concept of duty of care. Indeed, because community sport is largely run by volunteers, legal considerations have the potential to be opaque, or at least fail to address the nuances involved. For example, the uncertainty around what constitutes a workplace issue potentially leads to severe implications for CSCs and NSOs and could trigger a significant change to the sport industry. The pressure on one party over another to create a safe working environment only creates confusion about who ultimately owes the duty of care, especially where a volunteer is concerned. Without further research and steps to address the paradox, any measures adopted from a policy and governance perspective are likely to be reactionary and not afford volunteers with the protection common to paid employees. While the current arrangements appear to work in the delivery of Australian sport, it is envisaged that one major legal decision has the potential to create significant, costly, change to the sport industry.

Going forward, in the first instance, further research is needed to understand the impact of top-down policy on CSCs, but crucially also on volunteer experiences. There have been historical calls for investigations into sport governance in Australia (126), with more recent requests for research into government policy on community sport and volunteering (1), along with the establishment of research paradigms specific to sport volunteer management (127, 128). Based on real-world governance issues, it is important to stress that CSCs—policy implementers—are cognisant of the problems of unclear and ultimately top-down policy directives (16, 46, 56, 124). Although there has been research that points to the potential for greater autonomy for CSCs [e.g., (47)], evidence suggests that, at grassroots level, CSCs are some way from being in a position to properly manage fundamental aspects of organisational operations (47) and are generally at risk from a legal perspective (14).

Thus, based on the evidence, the element of risk to a sporting organisation warrants action to mitigate the problem. Such action might include (a) outlining the full extent of legal issues impacting CSCs and (b) providing avenues for cost and time effective training for CSCs on a national scale. In completing the objective at (a), the opportunity to fully understand the prevalence of risks to CSCs would be achieved, along with the potential to identify themes. In addition, there would be a greater likelihood of informing policy and governance protocols impacting stakeholders like CSCs. Albeit a significant resource issue, the suggestion at (b) must be considered crucial to equip CSC members with the skillsets necessary to operate as effectively as possible. Such action would help create an environment of “good governance” (8, 41) and provide a platform for better compliance procedures.

A more immediate step to consider, to develop levels of compliance and to alleviate the implications of litigation, relates to indemnities for CSCs. Insurance is vital for a CSC to protect itself, its office bearers, and its assets, and it represents a standard risk management tool, which may be used to reduce liability for CSCs. There are various potential hazards that CSCs may be exposed to, including: negligence; criminal liability; discrimination and harassment; occupier's liability; WHS; and employment liability. To mitigate such risks, CSCs need to enter into robust insurance policies, including public and employer liability insurance, a process that is complicated (129).

There are also issues with the cost of insurance for CSCs, which can impact a CSC's ability to secure adequate levels of insurance (130). Proactively pursuing suitable insurance coverage represents a key factor that assists CSCs in becoming more resilient (131). Still, in an ever-changing landscape, there are risks associated with insurance and maintaining resilience to the vicissitudes of community sport (132). Overall, for CSCs, insurance represents a challenging area to navigate and is a reactive measure rather than a practical and pre-emptive risk management tool.

## Conclusion

This paper identified numerous concerns impacting sport organisations—CSCs in particular—and outlined the irony that sport volunteers are unlikely to be able to fulfil expectations from a legal and governance perspective, thus necessitating a different outlook. The evidence presented herein clearly highlights significant legal concerns, signifies inadequacies in governance frameworks, and highlights a quandary for NSOs and arguably the most important asset to sport: volunteers. Issues relating to duty of care, terms of employment, and general liability were raised but ultimately created more questions than answers. Thus, to address the problems, thematic research into legal issues impacting community sport is needed, along with a greater and more cost-effective means for training volunteers running CSCs. Further, a more robust and user-friendly approach to insurance is required to adequately cover CSCs and their members. Overall, given the importance of community sport to the Australian nation and the fact that volunteers are essential to manage CSCs, significant steps are needed to address issues impacting volunteer engagement in sport and eliminate the associated paradox.
